# Identification of key genes with prognostic value in gastric cancer by bioinformatics analysis

**DOI:** 10.3389/fgene.2022.958213

**Published:** 2022-08-30

**Authors:** Rongsheng Wang, Xiaohong Chen, Cuilan Huang, Xiaogang Yang, Huiwei He, Chenghong OuYang, Hainan Li, Jinghua Guo, Chunli Yang, Zhiying Lin

**Affiliations:** Jiangxi Provincial People’s Hospital, The First Affiliated Hospital of Nanchang Medical College, Nanchang, Jiangxi, China

**Keywords:** gastric cancer, bioinformatics, key genes, protein–protein interaction network, Cytoscape

## Abstract

**Background:** Gastric cancer (GC) is a digestive system tumor with high morbidity and mortality. It is urgently required to identify genes to elucidate the underlying molecular mechanisms. The aim of this study is to identify the key genes which may affect the prognosis of GC patients and be a therapeutic strategy for GC patients by bioinformatic analysis.

**Methods:** The significant prognostic differentially expressed genes (DEGs) were screened out from The Cancer Genome Atlas (TCGA) and the Gene Expression Omnibus (GEO) datasets. The protein–protein interaction (PPI) network was established by STRING and screening key genes by MCODE and CytoNCA plug-ins in Cytoscape. Functional enrichment analysis, construction of a prognostic risk model, and nomograms verify key genes as potential therapeutic targets.

**Results:** In total, 997 genes and 805 genes were related to the prognosis of GC in the GSE84437 and TCGA datasets, respectively. We define the 128 genes shared by the two datasets as prognostic DEGs (P-DEGs). Then, the first four genes (*MYLK*, *MYL9*, *LUM*, and *CAV1*) with great node importance in the PPI network of P-DEGs were identified as key genes. Independent prognostic risk analysis found that patients with high key gene expression had a poor prognosis, excluding their age, gender, and TNM stage. GO and KEGG enrichment analyses showed that key genes may exert influence through the PI3K-Akt pathway, in which extracellular matrix organization and focal adhesion may play important roles in key genes influencing the prognosis of GC patients.

**Conclusion:** We found that *MYLK*, *MYL9*, *LUM*, and *CAV1* are potential and reliable prognostic key genes that affect the invasion and migration of gastric cancer.

## Introduction

Gastric cancer (GC) is the fifth most common cancer and the third most common cause of cancer-related deaths in the world. The statistical results showed that there were more than one million new cases of GC in the world every year, and the number of GC-related death cases continuously increased; statistics for 2018 showed that the death toll had risen to 784,000 (Smyth et al., 2020). Many interfering factors can cause the low survival rates of GC patients, among which the diagnosis of GC patients usually occurs in the middle and late stages; easy recurrence and metastasis after an operation are the most common reasons for the poor prognosis of GC patients (Fang et al., 2020). In the past 10 years, a large number of studies have revealed that there were quite sensitive and effective biomarkers that can affect the occurrence and progression of GC, for example, Graziano et al. (2004) found that methylation of the CpG island in the promoter region of the *CDH1* gene will lead to a change in CDH1 expression, which may play an important role in the occurrence and progression of diffusive GC, and *CDH1* is likely to be one of the therapeutic targets of GC. Several previous studies (Digklia and Wagner, 2016) also found that HER2 expression is not only an independent risk factor affecting the prognosis of GC patients but also an effective target for the treatment of GC patients. These experiences provide the basis for the research on the occurrence, progression, and treatment of GC. However, previous studies on biomarkers on the occurrence and progression of GC were based on a single-gene pattern, and cancer is usually a disease involving multiple genes and mechanisms. Therefore, it is very important to comprehensively explain the specific mechanism of GC progression and identify significant biomarkers to improve the prognosis of GC patients.

Bioinformatics is a broad multidisciplinary field. Computational tools have been developed to analyze and manage the increasing amount of biological data (Goujon et al., 2010). Bioinformatics can be used to identify the key drivers of each specific cancer patient. Therefore, they have the potential to realize more personalized cancer treatment programs, paving the way for new targeted drugs targeting specific proteins (Zhang et al., 2009). With the development of The Cancer Genome Atlas (TCGA), Gene Expression Omnibus (GEO), and the accumulation of cancer genetics knowledge that has developed rapidly in the last 10 years, tumor analysis based on databases not only reveals the whole panorama of tumor-related genome changes but also lays a foundation for the study of related tumor types (Cancer Genome Atlas Research et al., 2013).

In this study, bioinformatics methods and techniques were used to screen out prognostic differentially expressed genes (P-DEGs) of GC from GEO and TCGA databases. Furthermore , we established a PPI network to identify the key genes in DEGs through module analysis and centrality analysis, constructed a prognostic risk model, and verified an unfavorable indicator. This study provides a reliable basis for exploring the molecular mechanisms of GC pathogenesis and identifying molecular targets for clinical diagnosis or treatment.

## Methods

### Data

The gene expression matrix data on GC patients were obtained from the dataset (GSE84437) in the GEO database of the national bioinformatics center of the United States. The data set was composed of the gene chip expression profile data and the survival information on 433 GC patients, which were collected through the GPL6947 chip platform. Moreover, 380 cases of GC tissue expression profile data and clinical information were downloaded from TCGA database.

### Screening of prognosis-related genes of GC patients

The gene expression matrix of GC tissues was obtained from the GEO (n = 433) and TCGA (n = 380) databases, respectively, and then, the data were mined through R software. To obtain the standardized gene expression matrix of GC patients, the “impute” and “limma” packages in R were used to process the missing value estimation and logarithmic transformation of data. According to the K–M method, each gene in the gene expression matrix was divided into high- or low-expression groups based on the median value of the gene expression. Subsequently, the survival difference between these two groups was evaluated and verified. The proportional hazards model was used for multivariate analyses and survival estimation to analyze, verify, filter, and screen out these genes, which were significantly correlated with the prognosis of GC patients (*p* < 0.05). Finally, these genes filtered by the aforementioned survival analyses were mutually verified in the two datasets GEO and TCGA. Then, the common significant prognostic differentially expressed genes were identified as P-DEGs.

### PPI network

STRING (http://string-db.org) is an online tool, which is often used to predict protein–protein interactions (Szklarczyk et al., 2011). Through STRING, gene interaction analysis can be conducted, including physical and functional interactions. In this study, we used it to establish a PPI network of P-DEGs, while the confidence score of connections in this network is required to be >0.15, and the disconnected nodes in the network were excluded.

### Module analysis and centrality analysis of the PPI network

The PPI network of P-DEGs is visualized by Cytoscape software. In this network, the functional modules and the interactions between genes were identified and measured through the MCODE plug-in (Bader and Hogue, 2003). In all sub-modules, the higher the score was, the stronger the protein correlation in the sub-module was, and the sub-module with the highest score was considered the result of MCODE analysis. The plug-in CytoNCA is used for centrality analysis, including three parameters: degree, betweenness, and eigenvector (Tang et al., 2015). Degree is a measurement of the importance of a single node, which describes the number of sides of a connected node (Luo et al., 2017). Betweenness is the shortest path to analyze a specific node (Li et al., 2017). However, for the eigenvector, the importance of the node itself and its neighbors is considered (Negre et al., 2018). The top 5% of the nodes under each parameter are considered the important nodes of CytoNCA analysis, and the genes represented were considered as the result of centrality analysis. Finally, by combining the results of MCODE and CytoNCA plug-ins, the common genes were considered the most important genes in the PPI network of P-DEGs and identified as key genes.

### Prognostic analysis and validation of key genes

Based on the gene expression matrix data on GC patients in the GEO and TCGA databases, the median of key gene expression value was set as the cut-off value, and the key gene expression matrix of GC patients was divided into key gene high- and low-expression groups. By using the “survival” package in R, according to K–M analysis and a multivariate Cox regression test, the difference in overall survival events between the high- and low-expression groups of key genes was compared. Then, the survival rate and survival curve were analyzed and drawn. By using the “survival” package, according to univariate and multivariate Cox regression analyses, the hazard ratio (HR) and forest maps of independent prognostic analysis of single-gene and multiple-gene combinations of key genes were analyzed and drawn. Finally, to precisely predict the survival rates of GC patients, the risk scores of key genes and some clinicopathological factors, such as age, gender, and pathological stage, were linked together. According to the risk ratio-weighted key gene expression data, the key genes (Lin T. et al., 2018) were constructed as follows:

$$risk\;score=\sum_{i}^{N}\left(exp_{i}*HR_{i}\right),$$

where N is the number of selected genes of key genes, exp_*i*_ is the expression value of each single gene of key genes, and *HR*_*i*_ *i*s the HR value of each single gene in the multivariate Cox regression model. According to the median value of the risk scores of key genes in the expression matrix of GC patients, GC patients were divided into the low-risk group and the high-risk group, and the prognostic risk rates were measured by K–M analysis. Subsequently, based on the multivariate Cox regression analysis, the nomogram is established and drawn through the “RMS” package in the R language.

### GO and KEGG analyses of key genes

According to the median value of each key gene, GC patients in the GEO and TCGA databases were divided into high- and low-expression groups for each gene, respectively. The differentially expressed gene (DEG) sets between high- and low-expression groups of each key gene were identified, and the corresponding GO and KEGG functional enrichment analyses of each DEG were conducted through “limma,” “clusterProfiler,” “org.Hs.eg.db,” “enrichplot,” and “ggplot2″ R software packages. |log2FC|>0.5, *p* < 0.05, and adjusted *p* < 0.05 were considered as the cut-off criteria.

### Statistical analysis

R language (version 4.0.1) was used for data statistical analysis: K–M analysis, univariate Cox regression analysis, and multivariate Cox regression analysis were used to identify the key genes. Survival curves and forest maps of survival analysis and independent prognostic analysis of single-gene or multiple-gene combinations of key genes were drawn with the R language through the “survival” package. *p* < 0.05 and adjusted *p* < 0.05 were considered as the cut-off criteria.

## Result

### Identification of P-DEGs

**FIGURE 1:**
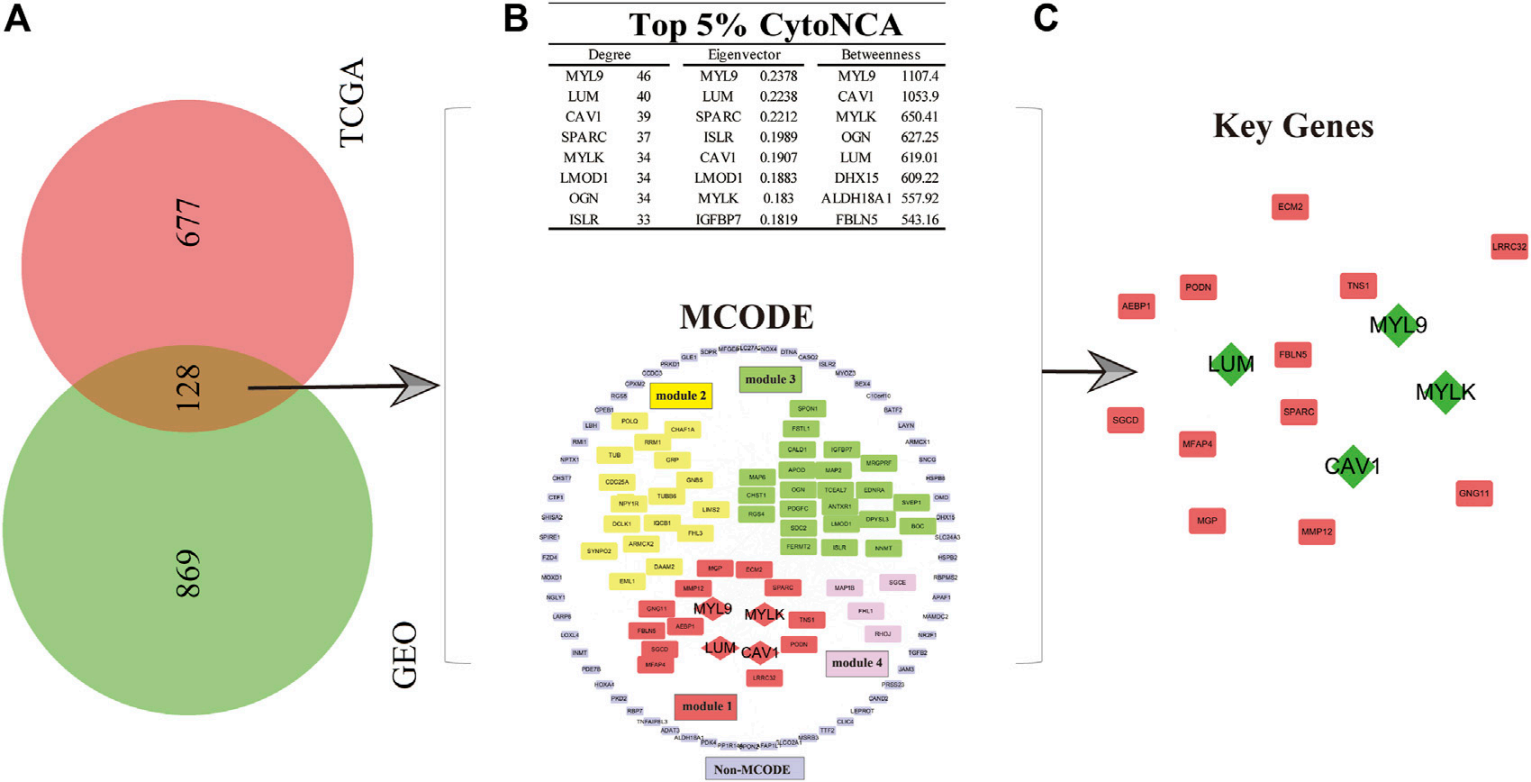
Selection of key genes for GC patients. **(A)** In total, 128 common P-DEGs were obtained from the intersection of TCGA and GEO datasets. **(B)** Four modules, namely, modules 1–4, and one non-MCODE module and a score ranked up in the top 5% in three parameters from CytoNCA’s centrality analysis. **(C)** Key genes (*MYLK*, *MYL9*, *LUM*, and *CAV1*, green diamond in the picture) were obtained.

To explore the key genes affecting the prognosis of GC patients and the roles these genes play in the mechanism of GC progression, the gene expression matrix data obtained from the GEO and TCGA databases were used to conduct multivariate analyses and survival estimation to screen out the genes that were significantly correlated with the prognosis of GC patients (*p* < 0.05). Subsequently, we obtained 997 and 805 genes related to the prognosis of GC in the GSE84437 and TCGA gene expression matrix datasets, respectively. Therefore, 128 common P-DEGs were obtained by mutual validation between the two datasets, which means 128 of 997 genes in GSE84437 and 128 of 805 genes in TCGA databases (Figure 1A).

### Module analysis and centrality analysis of the P-DEG-related PPI network

In order to study the molecular mechanism which can affect the prognosis of GC patients from a systematic perspective, we established a PPI network of P-DEGs to explore the molecular mechanism. The results showed that there were 124 nodes and 819 edges in the PPI network. Furthermore, we used the MCODE plug-in in Cytoscape software to analyze the modules available for exploring more closely related genes in the PPI network. The results showed that there were four modules and one non-module in the PPI network, and the scores of the four modules were as follows: 8.667 (module 1), 7.455 (module 2), 4.111 (module 3), and 2.667 (module 4), respectively. We found that the first module (module 1) was the most interactive area in the PPI network, which is located at the center of the whole network, including 16 nodes and 65 edges (Figure 1B). Therefore, the protein interactions in module 1, which rank the first, maybe the strongest and most important part of the whole network. The results of module 1 were considered the final result of the MCODE analysis. At the same time, to obtain GC prognosis-related key genes in this complex PPI network, we used the centrality analysis method to analyze the PPI network. First, we used the CytoNCA plug-in to analyze the score of three parameters of each gene in the PPI network, which were degree, betweenness, and eigenvector. Then, we selected the genes whose scores ranked in the top 5% in three parameters. Finally, we selected these genes which ranked top 5% in three parameters and showed up in module 1 as key genes, which were *MYLK*, *MYL9*, *LUM*, and *CAV1*, and they were all in module 1 with high centrality (Figure 1C).

### Prognostic value of key genes in GC patients

**FIGURE 2:**
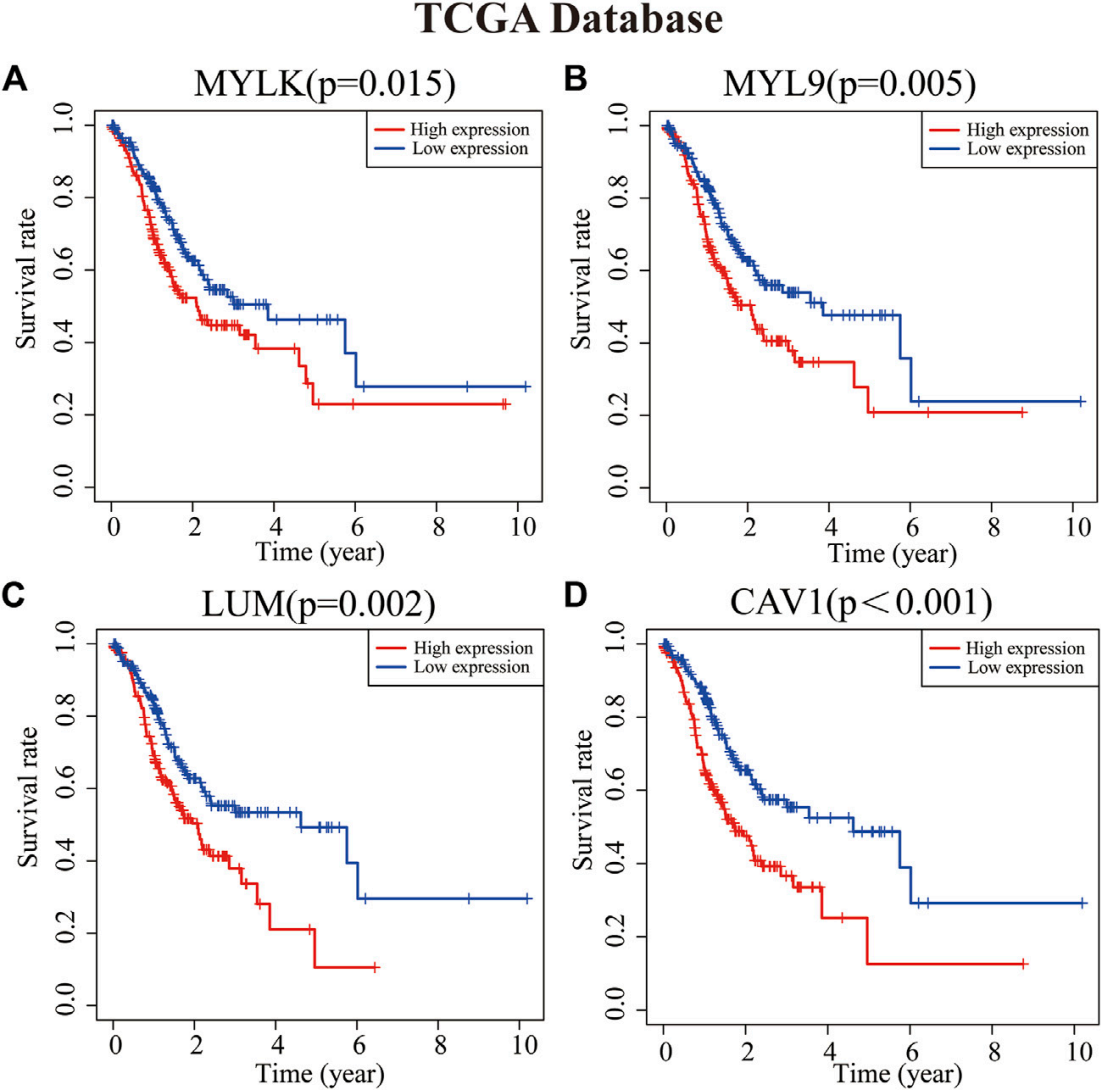
Survival analyses of key genes [**(A)** *MYLK*, **(B)** *MYL9*, **(C)** *LUM*, and **(D)** *CAV1*]. Patients with high expression of key genes have a poor prognosis (*p* < 0.05).

**FIGURE 3:**
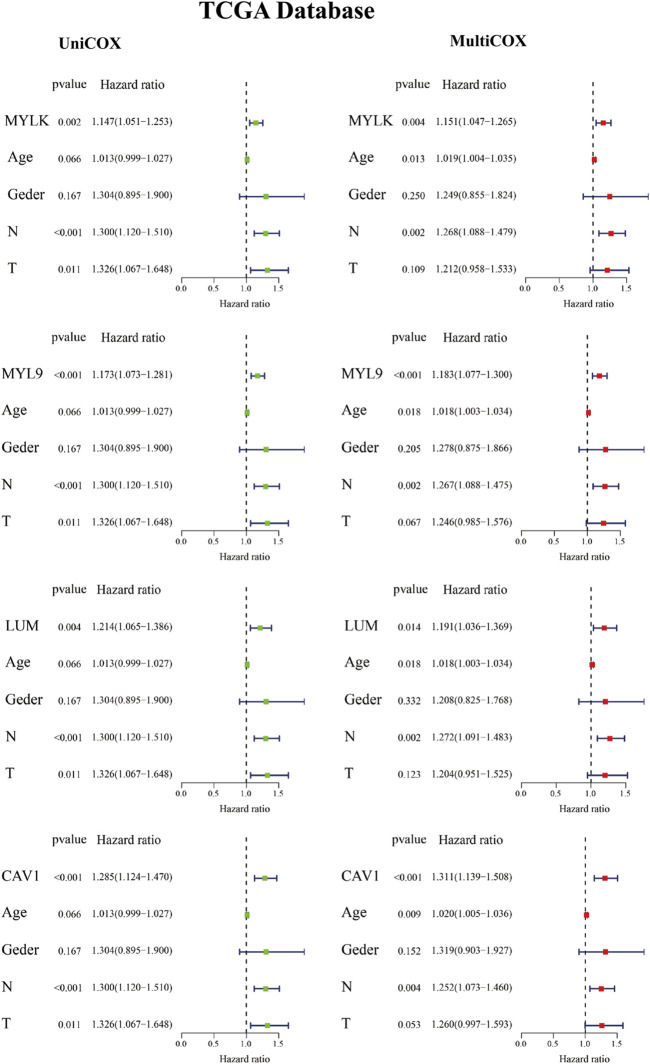
Independent prognostic analysis of key genes in TCGA database.

To analyze the role of key genes in the progression of GC, the survival analyses of four genes of key genes were further analyzed through the K–M method. According to the median expression of the gene matrix, GC patients were divided into the high-expression group and the low-expression group. The survival curve showed that the expressions of *MYLK*, *MYL9*, *LUM*, and *CAV1* were significantly correlated with the survival rate and overall survival time of GC patients in GEO and TCGA databases (*p* < 0.05). According to the survival analyses, the median survival time of GC patients with lower expression of *MYLK*, *MYL9*, *LUM*, and *CAV1* was1.37, 1.41, 1.35, and 1.42 years; with higher expression of *MYLK*, *MYL9*, *LUM*, and *CAV1*, the median survival time was 1.06, 1.08, 1.15, and 1.06 years in TCGA database, respectively. Compared with GC patients with lower expression of *MYLK*, *MYL9*, *LUM*, and *CAV1* (GEO, n = 217; TCGA, n = 190), these patients with high expression of key genes (n = 216, GEO; n = 190, TCGA) had significantly poorer prognosis (*p* < 0.05, Figures 2A–D and Supplementary Figures S1A-D). The results were verified through the GEO gene matrix once again. According to the univariate and multivariate Cox regression analyses, the results of independent prognosis of key genes in the GEO and TCGA databases showed that the HR of *MYLK*, *MYL9*, *LUM*, and *CAV1* were all presented as HR > 1, which were 1.15, 1.18, 1.19, and 1.31, respectively (*p* < 0.05). These results indicate that key genes can independently affect the prognosis of GC patients (Figure 3 and Supplementary Figure S2). The influence of key genes is of great significance and has potential value as prognostic biomarkers and therapeutic targets for GC patients.

### GO and KEGG enrichment analyses

**FIGURE 4:**
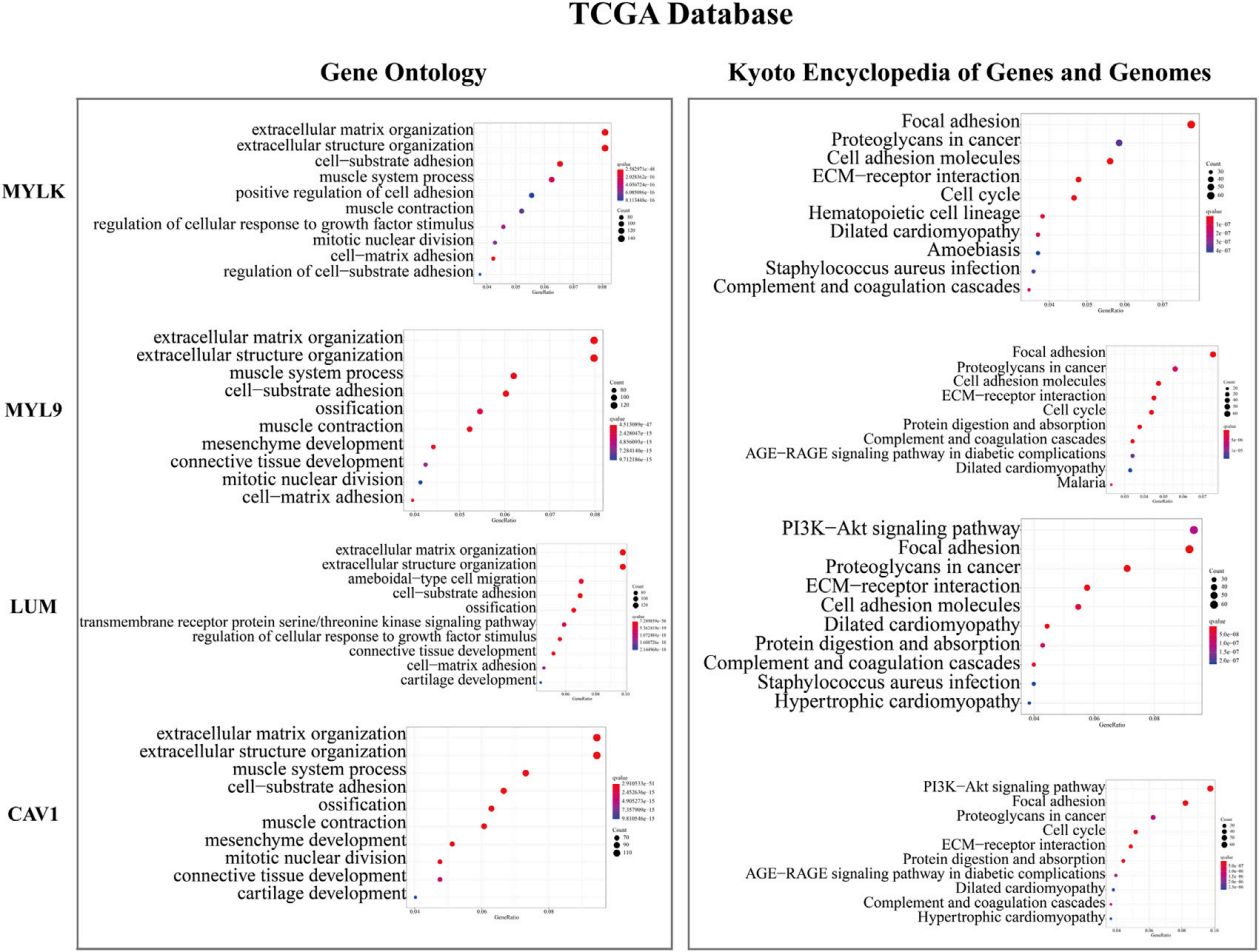
GO enrichment and KEGG enrichment analyses of key genes in TCGA database.

To better elucidate the mechanisms of key genes affecting GC prognosis, we performed GO and KEGG enrichment analyses. Results of GO analyses showed that most GO terms were significantly enriched in extracellular matrix organization, extracellular structure organization, cell-substrate adhesion, tissue migration, muscle contraction, muscle tissue development, mesenchymal development, etc. (Figure 4 and Supplementary Figure S3). Moreover, the results of KEGG analyses showed that the related pathways were significantly enriched in focal adhesion, PI3K-Akt signaling pathway, ECM receptor interaction, cell adhesion molecules, proteoglycans in cancer, protein digestion and absorption, cell cycle, calcium signaling pathway, etc (Figure 4). These results indicate that key genes affect the prognosis of GC patients mainly through influencing the invasion, migration, and cell cycle functions of GC cells.

### Construction and validation of the prognostic risk model of key genes

**FIGURE 5:**
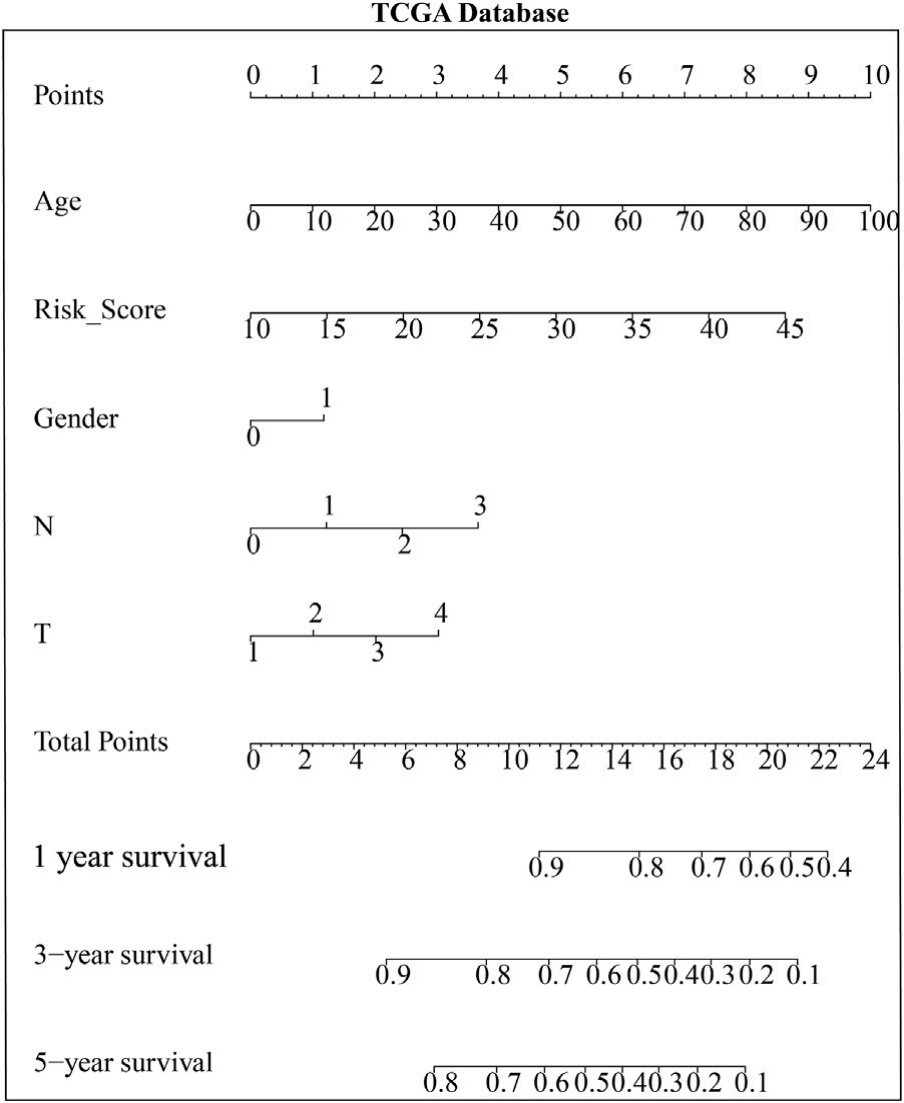
Nomogram based on the risk model and clinicopathological factors in TCGA database.

Based on multivariate Cox regression analysis, key genes (*MYLK*, *MYL9*, *LUM*, and *CAV1*) were integrated, and a prognostic risk model of key genes was established according to GEO and TCGA data, respectively. The risk scores of key genes were calculated using the formula mentioned in the method, and processes were as follows: risk score = (HR _(MYLK)_ × MYLK expression level) + (HR _(MYL9)_ × MYL9 expression level) + (HR _(LUM)_ × LUM expression evaluation rate, risk score, and clinical features of GC patients can be estimated based on the total points) (Supplementary Table S1). To confirm the prognostic value of the risk signature, we constructed a nomogram based on the prognostic risk model, and we determined the clinical relevance and prognostic value of age, gender, and TNM staging. The 1-year, 3-year, and 5-year survival rates can be estimated from the total scores, which are the sum of the scores for each item, as shown in the nomogram (Figure 5 and Supplementary Figure S4). The nomogram not only proved that the prognostic risk model is reliable but also showed that the accuracy of predicting survival in each patient was high.

**FIGURE 6:**
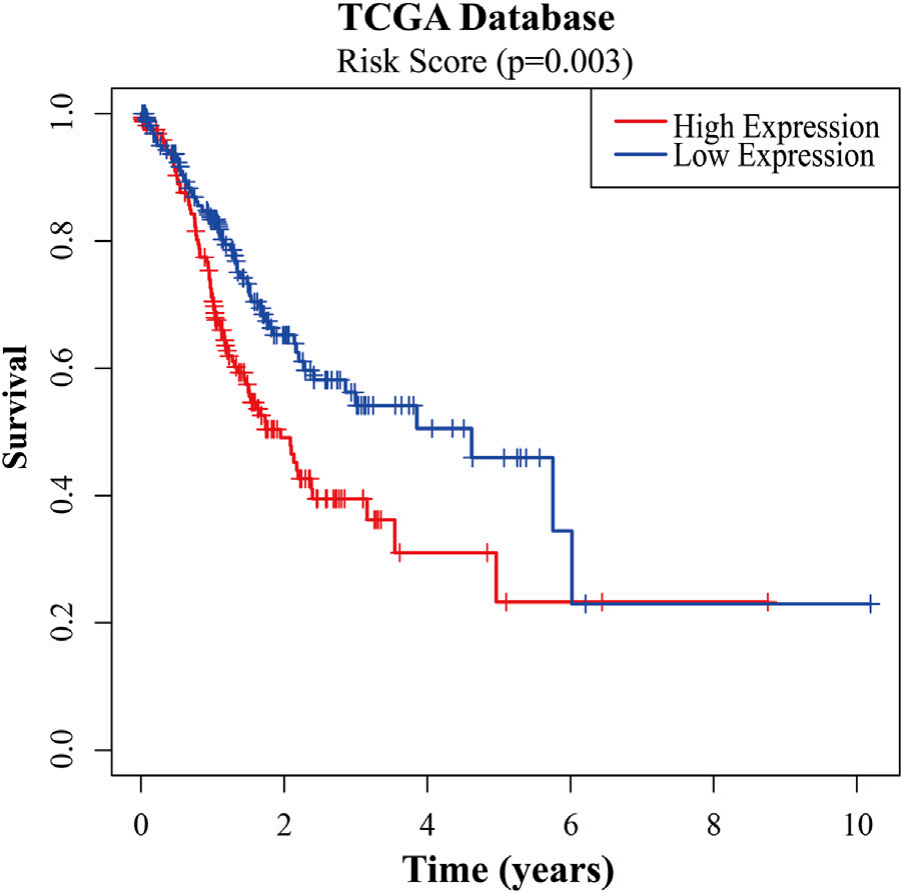
Survival analyses of the risk scores of key genes in TCGA database.

To further verify the reliability of key genes, GC patients were divided into the low-risk group and the high-risk group according to the median risk score in TCGA and GEO databases, respectively. The survival curves showed that the prognosis of the high-risk group was worse than that of the low-risk group (Figure 6, *p* < 0.05). With the risk score increasing, the number of patients’ deaths increases (Figure 6 and Supplementary Figure S5). Univariate and multivariate Cox regression analyses were performed based on the gene matrix data, the results of which showed that the risk scores of key genes were independently correlated with the overall survival rate of GC patients (Table 1, *p* < 0.05). These results indicate that the key genes can be a significant reference to the prognosis of GC patients. The key genes can be used to guide the next step of treatment after surgery or/and chemoradiotherapy treatment. *MYLK*, *MYL9*, *LUM*, and *CAV1* can be potential targets to improve the prognosis of GC patients.

**TABLE1:** Univariate and multivariate analyses of the prognostic risk model in TCGA database.

Variable	Univariate analysis		Multivariate analysis	
	HR	*p*-value	HR	*p*-value
Risk score	1.0464	0.0003	1.0489	0.0004
Age	1.0129	0.0659	1.0196	0.0120
Gender	1.3037	0.1674	1.2572	0.2364
T	1.3004	0.0006	1.2674	0.0023
N	1.3263	0.0109	1.2162	0.1035

## Discussion

GC is one of the most common and malignant tumors. Although the main treatment methods for GC such as surgery, radiotherapy, and chemotherapy have made progress, the incidence rate and mortality rate of GC patients remain stubbornly high (Ferlay et al., 2015; Li et al., 2020). More than 90% of the GC patients were in the late stage when diagnosed, which was related to the unclear symptoms in the early stage of GC patients and unclear influential factors of GC prognosis to a large extent (Yan et al., 2018; Huang et al., 2019). The occurrence and progression of GC is a multi-stage, slow-moving pathological process, in which genetic mutations, epigenetic changes, and abnormal molecular signal transduction pathways can all participate in the occurrence, diffusion, and metastasis of GC (Shan et al., 2019). Therefore, it is very important to find specific prognostic biomarkers of GC to develop therapeutic strategies for malignant behaviors of tumors. These problems highlight the necessity of finding prognostic markers for GC. Nowadays, high-throughput platforms for detecting gene expression have been developed rapidly in the processes of disease progression, which lays the foundation for the discovery of new targets that can be used to predict, diagnose, and treat cancer.

Module analysis (MCODE) and centrality analysis (CytoNCA) in the PPI network play important roles in screening molecular markers; these genes appear in the modules with the highest scores and also rank higher in centrality analysis results, which are the key genes that can affect the occurrence of diseases (Tang et al., 2015). Studies have shown that module analysis can help screen key genes in cancers more accurately, such as cervical cancer (Xia et al., 2018), glioblastoma (Yang et al., 2018), and head and neck squamous cell carcinoma (Yang et al., 2017). However, CytoNCA can analyze the centrality degree of each node in the whole PPI network and can exhibit the nodes with important connections, to help select key genes (Lu et al., 2019). Combined with these two methods, key genes (*MYLK*, *MYL9*, *LUM*, and *CAV1*) with important value in the whole PPI network were obtained. Some studies also elucidated the impact of key genes on various tumors.

Liang X et al. indicated that caveolin 1 (CAV1) plays an important role in the occurrence and progression of varieties of malignant tumors, especially in the malignant progression of GC, by promoting epithelial–mesenchymal transition (EMT) function. Under the conditions of the extracellular matrix integrin interaction and Tyr-14 phosphorylation, CAV1-enhanced melanoma cells will migrate, invade, and migrate to the lungs (Liang et al., 2018; Luo et al., 2020). Positive CAV1 expression is associated with progression and poor prognosis in GC patients after radical gastrectomy (Seker et al., 2017). The results of Jin et al. (2016) showed that, compared with normal gastric mucosa, myosin light chain 9 (MYL9) was abnormally upregulated in GC patients’ tumor tissues, and it could affect the prognosis of GC patients through adhesion plaque and leukocyte cross-endothelial migration. As an important part of the extracellular matrix, luminan (LUM) can be expressed in many organs and tissues of the human body. LUM can play an important role in tumor metastasis and invasion through extracellular matrix (Chen et al., 2020). The previous research study indicated that LUM could be regulated as a potential prognostic marker and therapeutic target for GC (Chen et al., 2020). Myosin light chain kinase (MYLK) can catalyze the phosphorylation of the myosin light chain and regulate the invasion and metastasis of some malignant tumors (Tan and Chen, 2014; Lin J. et al., 2018).

In the past few years, there has been more and more evidence of the key role of the extracellular matrix in mediating different cell processes (including cell adhesion, polarity, migration, differentiation, proliferation, and survival), and tumor cells are closely related to it (Moreira et al., 2020). Focal adhesion is a strong adhesion of the sub-cellular structure to the extracellular matrix. It also acts as a scaffold for many signal transduction pathways involving integral proteins or mechanical force exerted on cells (Burridge, 2017). Focal adhesion dysfunction is considered to be an essential pathway in tumor invasion and migration (Carragher and Frame, 2004; Paluch et al., 2016). Many cellular processes in cancer are attributed to kinase signaling networks. Akt, as a serine/threonine kinase, also known as protein kinase B, is a carcinogenic protein that can regulate cell survival, proliferation, growth, apoptosis, and glycogen metabolism. Over-expression of Akt is a common molecular feature of human malignant tumors. Many tumor tissues and tumor cells are accompanied by activation of the PI3K/Akt signaling pathway (Song et al., 2019). In this study, we explored the relationship between key genes and classical carcinogenic signaling pathways by GO and KEGG enrichment analyses. Results showed that key genes can promote the development of GC by regulating various signaling pathways, many of which have been proven to play important roles in the occurrence and progression of cancer. In particular, focal adhesion and PI3K/Akt signaling pathways may be the main signaling pathways involved in the effect of key genes on GC prognosis, and their influences cannot be divorced from the extracellular matrix.

In this study, we integrated GEO and TCGA databases, using bioinformatics analysis methods, to mine and analyze high-throughput data to conduct module and centrality analysis of the PPI network, which helped us screen out key genes (*MYLK*, *MYL9*, *LUM*, and *CAV1*) that have an important impact on the prognosis of GC patients and can be considered as a biomarker and potential therapeutic target for GC prognosis. Then, the establishment of a prognostic risk model of key genes further explained the kernel roles the key genes may play in the development of GC.

## Conclusion

The integrative analyses of the gene expression matrix identified 128 common P-DEGs. The four key genes (*MYLK*, *MYL9*, *LUM*, and *CAV1*) of P-DEGs may be predictive biomarkers or therapeutic targets for GC prognosis. These predictions should be verified through experimental validation, although this study provided new insights into the development of individualized therapeutic targets for GC.

## Data Availability

Publicly available datasets were analyzed in this study. These data can be found at: https://cancergenome.nih.gov/ and https://www.ncbi.nlm.nih.gov/geo/.
