# Supplementary figures and images for: Identification of key genes with prognostic value in gastric cancer by bioinformatics analysis
Source: Front Genet. 2022 Aug 30;13:958213. doi: 10.3389/fgene.2022.958213 (PMC9468639; doi:10.3389/fgene.2022.958213)

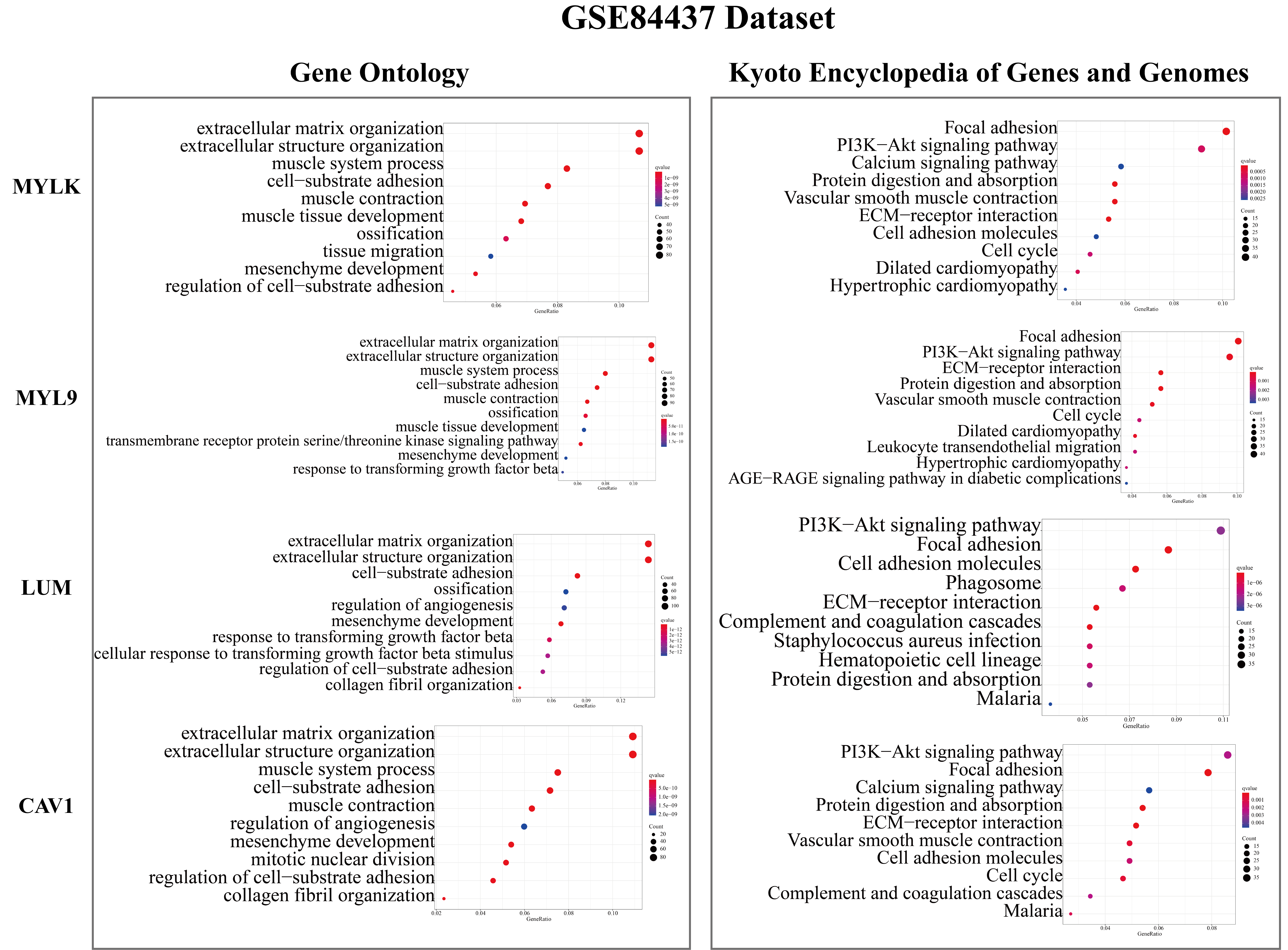

Supplement: Supplementary file 1 [file Image3.TIF]

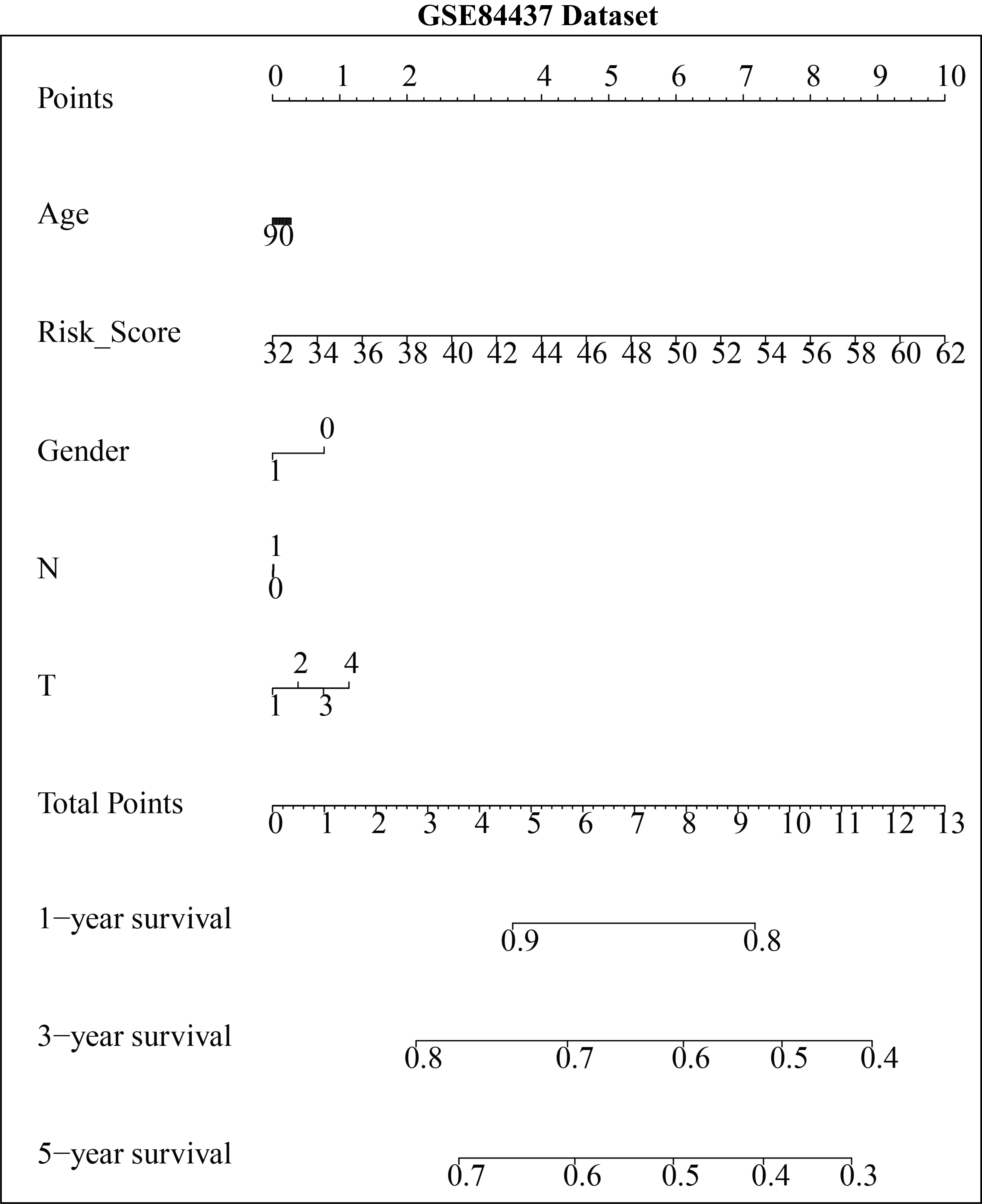

Supplement: Supplementary file 2 [file Image4.TIF]

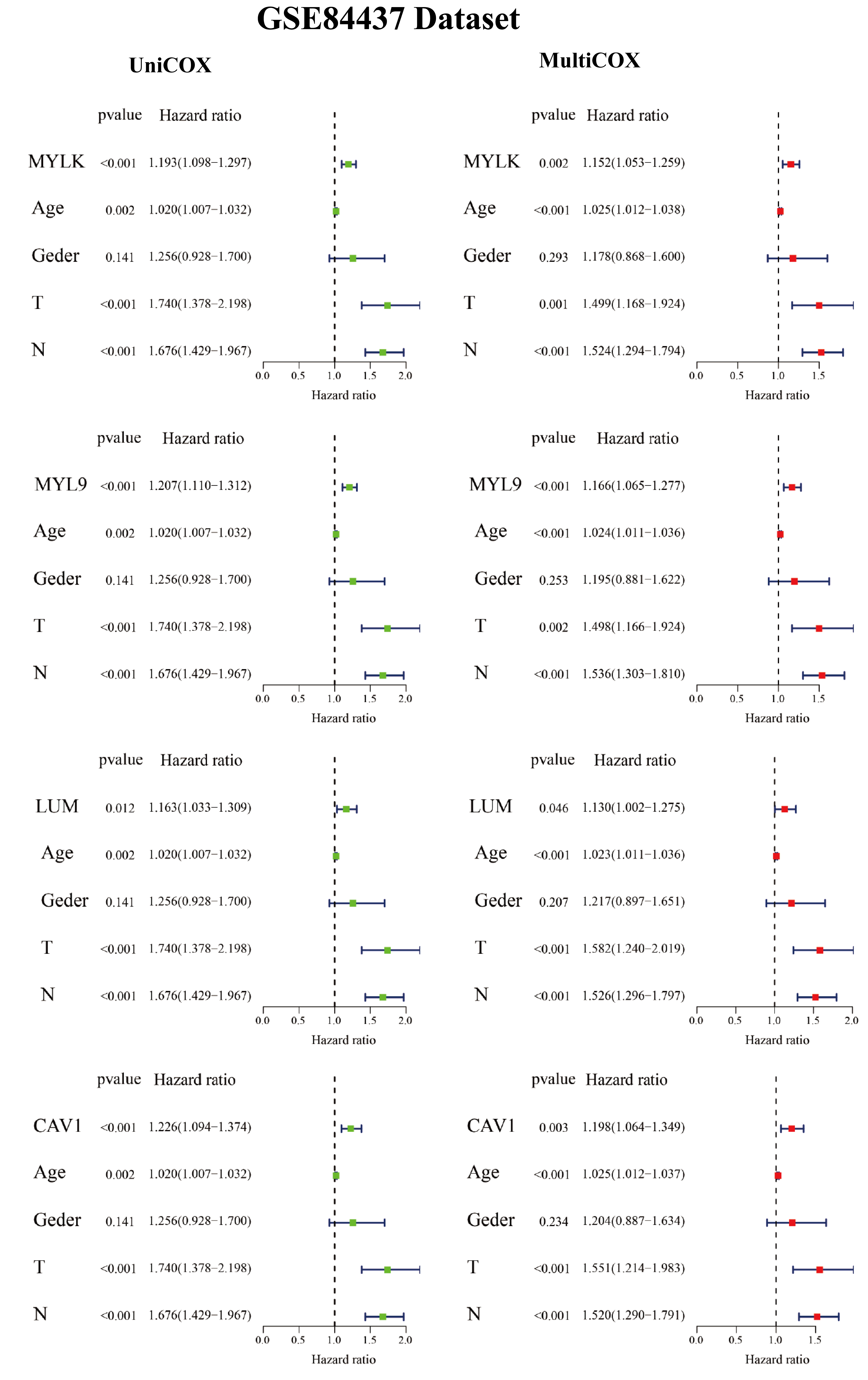

Supplement: Supplementary file 3 [file Image2.TIF]

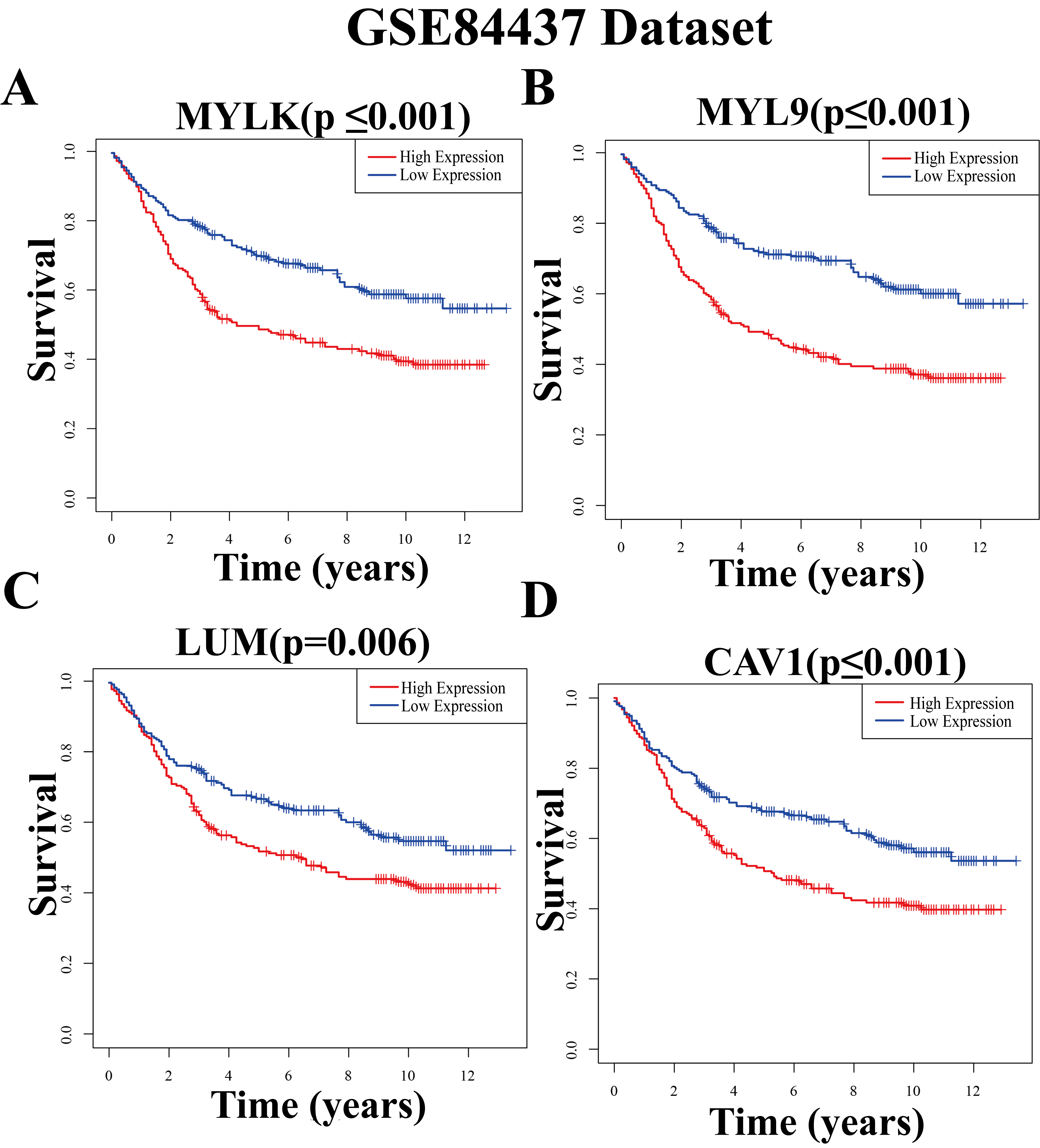

Supplement: Supplementary file 4 [file Image1.TIF]

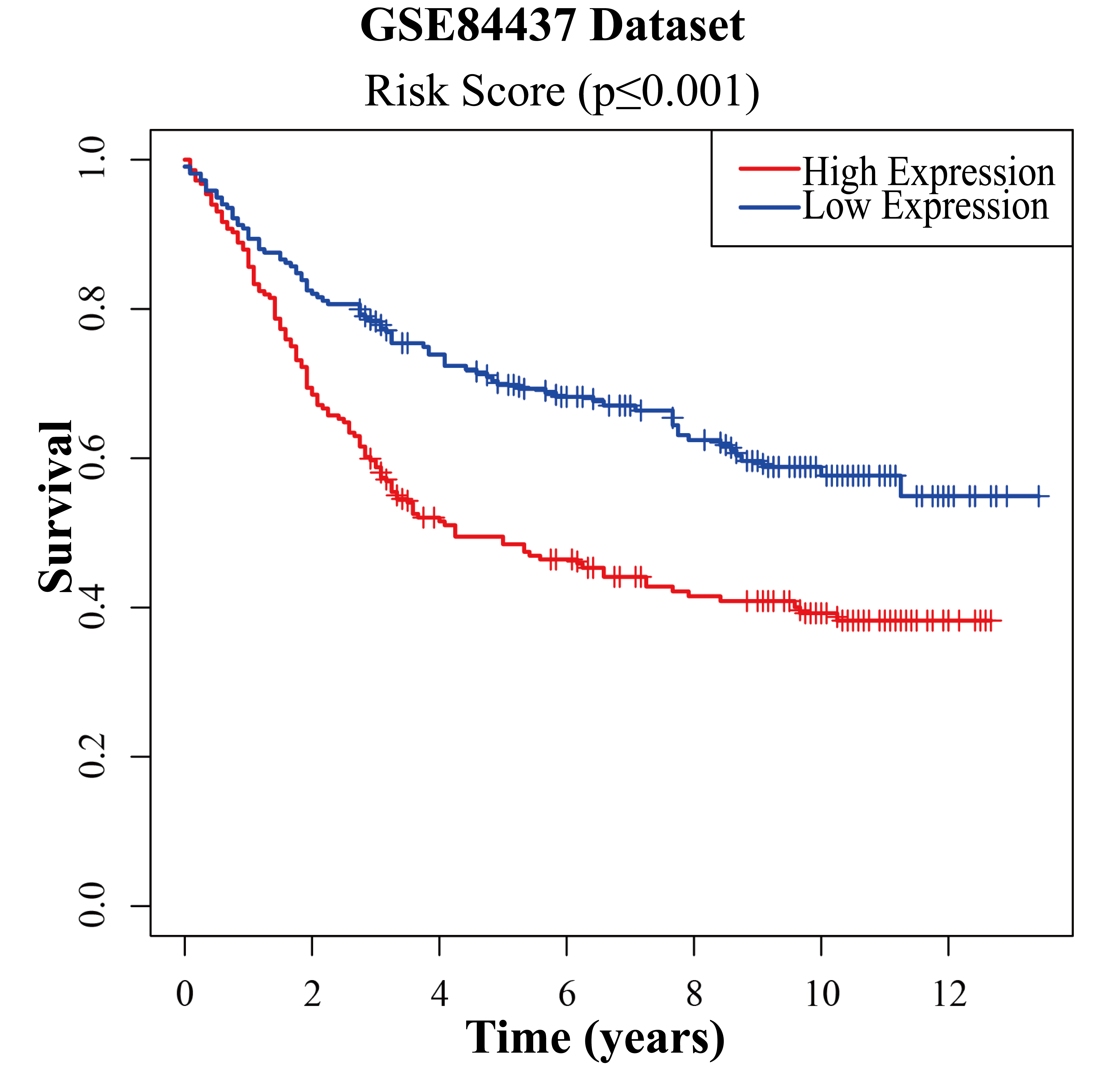

Supplement: Supplementary file 5 [file Image5.TIF]
